# The effects of developmental model of peer observation on the virtual teaching quality of basic medical sciences faculty

**DOI:** 10.1186/s12909-023-04331-z

**Published:** 2023-05-22

**Authors:** Mohammad Habibi Doroh, Arezou Farajpour, Elham Mokhtari Amirmajdi

**Affiliations:** 1 Otorhinolaryngology Head & Neck Surgery Department, Mashhad University of Medical Sciences, Mashhad, Iran; 2 Department of Basic Sciences, Faculty of Medicine, Mashhad Medical Sciences, Islamic Azad University, Mashhad, Iran; 3 Department of internal medicine, Faculty of Medicine, Mashhad Medical Sciences, Islamic Azad University, Mashhad, Iran

**Keywords:** Peer observation, Virtual education, Developmental model, Teacher evaluation

## Abstract

**Background:**

After the Corona pandemic, medical education has shifted to virtual education, but there has been limited time and possibilities for empowering faculty for this purpose. Therefore, it seems necessary to evaluate the quality of the provided training and provide feedback to the faculty in order to improve the quality of training. The purpose of this study was to investigate the effect of teacher formative evaluation by peer observation method on the quality of virtual teaching of basic medical sciences faculty.

**Methods:**

In this study, seven trained faculty members observed and based on a checklist evaluated the quality of 2 virtual sessions taught by each faculty of basic medical sciences, and provided them feedback; after at least 2 weeks, their Virtual teachings were again observed and evaluated. The results before and after providing feedback were compared through SPSS software.

**Results:**

After intervention, significant improvements were observed in the average scores of “overall virtual performance”, “virtual classroom management” and “content quality”. Specifically, there was a significant increase in the average score of “overall virtual performance” and “virtual class management” among female faculty, and the average score of “overall virtual performance” among permanently employed faculty members with more than 5 years of teaching experience, before and after intervention (p < 0.05).

**Conclusion:**

Virtual and online education can be a suitable platform for the implementation of formative and developmental model of peer observation of faculty; and should be considered as an opportunity to empower and improve the quality of the faculty’ performance in virtual education.

**Supplementary Information:**

The online version contains supplementary material available at 10.1186/s12909-023-04331-z.

## Background

Peer observation of teaching as a means of academic improvement has long been considered as a part of teacher evaluation or quality assurance systems of educational institutions [1, 2]. Since faculty are one of the main pillars of universities and their educational role has a direct impact on the quality of educating the future professionals, the evaluation and development of faculty’ performance has been always considered by managers and planners [3–9]. At present, peer observation of teaching is one of the factors to assure the quality of the educational system and one of the evaluation methods, which along with providing appropriate and constructive feedback can lead to improvements in educational quality [10, 11]. The goals of peer observation include strengthening professional growth, increasing teaching effectiveness, increasing communication between faculty members, creating means to provide constructive feedback, and identifying faculty who operate at a high level of teaching skills [12, 13]. Therefore, it is necessary to investigate the faculty’ response to peer observation and its impact on the quality of teaching and learning [10]. A major challenge of peer observation is related to controlling bias among observers, which is called positive and negative bias. Positive bias can occur when faculty themselves choose the observer and negative bias can occur when a limited number of peer observers are used. In addition, studies show that if the evaluations are based on observations over time and the observers are highly anonymous, it can control bias [11]. In addition to the bias among peer observers, face-to-face peer review has other potential drawbacks. As a result, when teachers are observed by observers, they may become anxious, thereby negatively affecting their performance, or conversely, they may be motivated to improve their performance. This is the weakness of many face-to-face observational studies. Additionally, another major challenge of such evaluations is the considerable time required for the peer observation process [14, 15].

After the corona pandemic crisis in the world, medical education suddenly shifted to electronic and online education, while there were limited time and opportunities to empower faculty in this field. One of the successful and effective ways to evaluate and empower faculty members is to use the capacities and experiences of faculty members themselves. Based on literatures, peer observation of teaching is a learning focused approach that leads to teacher development [13, 16]. Today, due to the increasing use of virtual education, it is necessary for teachers to share their capacities and experiences for the growth and promotion of each other in this type of education. Virtual education can be significantly different from face-to-face education. Therefore, in order to to help the growth of online learning, peer observation of teaching should be developed to enhance teaching skills in faculty members and facilitate the formation of rethinking and reflection in virtual teaching processes [17]. As bell (2005) explained Observation of peer teaching skill is done in three separate models with different goals: (a) Management model which is done with management goals of promotion and tenure among faculty members, (b) Developmental model in which medical education experts and specialists observe doctors and seek educational improvement by providing constructive feedback, (c) Peer review model in which faculty members observe and evaluate each other with the aim of participating in the improvement of their education and mutual interaction [18]. So during covid19 pandemic, virtual education and electronic platform provide suitable situation to blind peer observation of teaching and teaching skill development through constructive feedback. The present study aimed to investigate the efficacy of the use of peer observation for development. in the faculty of basic medical sciences in Mashhad Islamic Azad University of medical sciences.

## Method

This quasi-experimental study was conducted using the one group pretest-posttest design in the academic year of 2020–2021 at the Islamic Azad University of Medical Sciences, Mashhad Branch, Iran. The research community consists of the faculty of the basic sciences department of medical school. They affiliated school of medicine and all of them presented online didactic courses for medical students in autumn semester in 2020 and included through census.

Seven faculty members who had a master’s degree in medical education participated in this study as the peer observer team. This group was justified and trained about the objectives of the study, and the ways to observe and evaluate. The evaluation tool was a researcher’s evaluation checklist consisting of 22 items, designed based on the opinion of experts about the quality of virtual teaching in four areas of virtual classroom management (item 1–6), content quality (item 7–11), organizing interactions (item 12–17) and managing motivation (item 18–22). At the beginning of the academic semester, the faculty members were informed by the Education Development Center that the online class sessions will be randomly evaluated by a random peer and constructive feedback will be given; they were also informed that the results will be confidential, only given to the faculty himself, and are not used in making decisions about their job titles. Observers attended the online class with the identity of an unknown participant and evaluated the relevant class based on the checklist, and since the faculty and students were not aware of his presence, there was no interference in the normal course of the class. All online classes of basic sciences were included in the study based on the inclusion criteria. The entry criteria included all basic sciences faculty employed with any type of contract who taught specific theoretical courses, virtually. And the exclusion criteria were practical or clinical and other general non medical courses that were held virtually due to the Corona crisis.

Each observer was randomly responsible for observing two online class sessions of 2 faculty- two random sessions for each faculty- during the first half of the semester. Then, with the cooperation of the Education Development Center, the detailed observation report include examples of observation, evaluative and constructive feedback were confidentially sent to the respective faculty. At least two weeks after providing the faculty with the feedback, the evaluator observed another class session of the same faculty member, in the second half of the semester or in the next semester, and filled the relevant checklist again.

The average scores of two observations before providing feedback and the score of checklist after providing feedback were compared, moreover, the relation between before-after scores and demographic variable was investigated.

This project is approved by the Ethics Committee of Islamic Azad University, Mashhad Medical Sciences school, with the code of “IR.IAU.MSHD.REC.1400.047”. The principle of confidentiality was observed during this study. Peer observation was done as one of the strategies of the Education Development Center and all faculty were informed about it, at the beginning of the semester. The judgment of the observer was confidential, only the faculty was informed about it, and was not included in the career decisions. The presence of an online observer was imperceptible and did not interfere with educational activities. Finally, after completing and collecting the checklists and entering the data into the computer, the data was analyzed using SPSS version 20 software. First, the normality of the data was checked using the Kolmogorov-Smirnov test. In addition to the descriptive statistics, the significance of the relations between the variables was checked through the Paired t-test. The significance level of the tests was considered less than 0.05.

## Results

The total number of the basic sciences faculty was 28, out of which 8 taught general non-medical courses such as English language, Islam- Iran history etc. that presented by faculty not medical school affiliated and 3 taught practical courses like laboratory sections, so they were excluded from the study; 2 other faculty were also excluded due to the termination of cooperation with the university in the next semester, despite they had been under evaluation and formative feedbacks were provided to them, in the first semester.

In this study, a total of 45 online class sessions were observed and evaluated by peers. 15 faculty of basic sciences courses of the medical school with an age range of 36-71years and an average age of 50.0 ± 10.04 years were investigated, including 8 men (53.3%) and 7 women (46.7%). Among the studied faculty, the least experienced professor had 2 years of experience and the most experienced one, 41 years. The average teaching experience of the studied faculty was 11.73 ± 12.3 years. More than half of the faculty (53.3%) were employed with permanent contracts, 4 faculties (26.7%) with temporary contracts, and the rest 3 (20%) worked as hourly staff. Due to the normality of the virtual performance overall score, paired t-test was used to compare the pre-post mean scores. The average overall score of the faculty’s virtual performance before providing feedback was 57.63 ± 10.54 and after providing feedback, it was 64.13 ± 13.32; the results showed a significant difference between the average overall score of virtual performance before and after providing feedback. There is (t = 3.7, p = 0.002). There was a significant positive correlation between the overall scores of faculty’ virtual performance in the first two observations before giving feedback (p = 0.001, r = 0.9).

The average score of virtual classroom management before giving feedback was 19.23 ± 4.16 and after giving feedback it reached 21.46 ± 4.85, which is statistically significant (p = 0.01, t = 2.8).

The average score of the course content quality before providing feedback was 14.33 ± 3.75, while after providing feedback, it was 16.47 ± 4.85; a significant difference was found between the two mean scores (t = 2.7, p = 0.02).

The average score of organizing interactions before providing feedback was 13.36 ± 4.59 and after the feedback, it was 13.60 ± 5.73; the difference was not significant (t = 0.27, p = 0.79). The average score of motivation management in the virtual class before giving feedback was 10.70 ± 3.73 and it was 12.60 ± 4.88 after the feedback; however, this difference was not also statistically significant (t = 1.7, P = 0.1 ).

According to Table 1, the results revealed that only among female faculty, there was a significant difference between the average overall score of the faculty’s virtual performance and the virtual classroom management score before and after intervention (p < 0.05).

**Table 1:** The mean ± SD of the scores of the dimensions of the checklist before and after the intervention by gender

Gender	Score	Group	Mean ± standard deviation	The result of the paired t test (p-value)
Male	Overall virtual performance	pretest	56.44 ± 24.12	T = 2.07 P = 0.07
		posttest	63.00 ± 16.49	
	Virtual classroom management	pretest	18.25 ± 4.70	T = 1.57 P = 0.16
		posttest	20.25 ± 6.27	
	Quality of course content	pretest	14.63 ± 4.83	T = 2.24 P = 0.06
		posttest	15.87 ± 6.15	
	Interaction organization	pretest	13.13 ± 4.60	T = 0.30 P = 0.77
		posttest	13.50 ± 6.21	
	Motivation management	pretest	10.44 ± 3.48	T = 1.57 P = 0.16
		posttest	13.38 ± 4.50	
Female	Overall virtual performance	pretest	59.00 ± 8.96	T = 4.35 P = 0.005^*^
		posttest	65.43 ± 9.64	
	Virtual classroom management	pretest	20.35 ± 3.44	T = 2.70 P = 0.04^*^
		posttest	22.85 ± 2.19	
	Quality of course content	pretest	14.00 ± 2.31	T = 2.1 P = 0.08
		posttest	17.14 ± 3.13	
	Interaction organization	pretest	13.64 ± 4.93	T = 0.05 P = 0.97
		posttest	13.71 ± 5.61	
	Motivation management	pretest	11.00 ± 4.26	T = 0.80 P = 0.45
		posttest	11.71 ± 5.49	

As shown in Table 2, there is a significant difference between the average overall score of the faculty’s virtual performance before and after the feedback, only among the official faculty employed with permanent contracts (p < 0.05).

**Table 2:** The mean ± SD of the scores of the dimensions of the checklist before and after the intervention by type of cooperation

Type of cooperation	Component	Group	Mean ± standard deviation	The result of the paired t-test (p-value)
Permanent Contract	Overall virtual performance	pretest	61.19 ± 9.71	T = 4.50 P = 0.003*
		posttest	71.25 ± 9.09	
	Virtual classroom management	pretest	21.00 ± 3.54	T = 2.62 P = 0.04*
		posttest	23.13 ± 3.27	
	Quality of course content	pretest	16.63 ± 3.83	T = 0.61 P = 0.56
		posttest	17.87 ± 4.61	
	Interaction organization	pretest	14.37 ± 4.52	T = 2.20 P = 0.07
		posttest	15.38 ± 6.16	
	Motivation management	pretest	11.19 ± 3.96	T = 0.47 P = 0.60
		posttest	14.87 ± 4.22	
Hour work	Overall virtual performance	pretest	61.00 ± 12.13	T = 1.90 P = 0.19
		posttest	62.33 ± 16.44	
	Virtual classroom management	pretest	18.83 ± 1.15	T = 2.64 P = 0.11
		posttest	19.33 ± 2.51	
	Quality of course content	pretest	16.83 ± 1.26	T = 0.38 P = 0.74
		posttest	18.00 ± 1.73	
	Interaction organization	pretest	13.17 ± 7.84	T = 1.21 P = 0.31
		posttest	12.00 ± 8.19	
	Motivation management	pretest	12.17 ± 4.26	T = 0.65 P = 0.56
		posttest	13.00 ± 4.58	
Temporary Contract	Overall virtual performance	pretest	48.00 ± 5.61	T = 0.48 P = 0.66
		posttest	51.25 ± 9.78	
	Virtual classroom management	pretest	16.00 ± 5.30	T = 2.70 P = 0.07
		posttest	19.75 ± 7.59	
	Quality of course content	pretest	11.87 ± 3.94	T = 2.10 P = 0.08
		posttest	12.50 ± 5.44	
	Interaction organization score	pretest	11.50 ± 1.47	T = 0.05 P = 0.97
		posttest	11.25 ± 1.26	
	Motivation management score	pretest	8.63 ± 3.40	T = 0.80 P = 0.45
		posttest	7.75 ± 3.20	

As Table 3 demonstrates, only among faculty with more than 5 years of teaching experience, there is a significant difference between the average overall score of the faculty’s virtual performance before and after the intervention (p = 0.02).

**Table 3:** The mean ± SD of the scores of the dimensions of the checklist before and after the feedback by teaching experience

Teaching experience	Component	Group	Mean ± standard deviation	The result of the paired t-test (p-value)
5 years and less	Overall virtual performance	pretest	57.00 ± 13.60	T = 2.07 P = 0.07
		posttest	65.14 ± 15.00	
	Virtual classroom management	pretest	17.71 ± 3.92	T = 1.57 P = 0.16
		posttest	20.0 ± 5.51	
	Quality of course content	pretest	12.43 ± 3.77	T = 2.24 P = 0.06
		posttest	13.71 ± 4.95	
	Interaction organization	pretest	16.36 ± 5.99	T = 0.30 P = 0.77
		posttest	16.00 ± 5.16	
	Motivation management	pretest	12.50 ± 3.80	T = 1.57 P = 0.16
		posttest	15.40 ± 3.70	
More than 5 years	Overall virtual performance	pretest	58.20 ± 7.92	T = 3.27 P = 0.02*
		posttest	63.20 ± 12.59	
	Virtual classroom management	pretest	20.56 ± 4.13	T = 1.70 P = 0.14
		posttest	22.75 ± 4.13	
	Quality of course content	pretest	16.00 ± 3.02	T = 2.36 P = 0.06
		posttest	18.87 ± 3.44	
	Interaction organization	pretest	12.50 ± 3.07	T = 2.13 P = 0.08
		posttest	11.50 ± 5.65	
	Motivation management	pretest	9.12 ± 3.06	T = 2.25 P = 0.06
		posttest	10.12 ± 4.58	

## Discussion

This study aimed at investigating the effects of peer evaluation on the quality of virtual education of basic sciences faculty of Azad Medical School. In general, the results showed that there is a significant difference between the average score of virtual education performance of faculty before and after providing feedback. The average score of overall virtual education increased from 57.63 to 64.13 after intervention. To the best of our knowledge, no similar study was previously conducted inside the country and the few studies in this field are done abroad. In the study by Marcy E. Rosenbaum et al., published in 2005 in the American Department of Family Medicine under the title “Using peer evaluation system to assess faculty performance and competence”, a comprehensive faculty peer evaluation system was designed to assess the six competencies as well as faculty performance in their teaching. Using a one-page form containing 19 items, all faculty members evaluated all other faculty of the family medicine training department, annually. In their study, three main components were evaluated; they include “clinical practice and teaching”, “departmental citizenship” and “research”. The results of their study showed that regarding the measures related to role within the department, faculty who had primary administrative responsibility outperformed the other faculty. And research faculty surpassed others in “research skills’’; and no differences in subgroup scores for clinical skills were observed. Therefore, in this study, it was concluded that using a method in which all faculty evaluate each other can result in objective, reliable measures of faculty performance [11]. So, based on the findings of our study and other studies, it is estimated that Peer observation and evaluation can improve the performance of faculty, and as found in our study, from the point of view of peers, the quality of virtual education is increased after providing feedback.

Lee S. Eiland and his colleagues, 2020, at the Auburn School of Pharmacy published a paper entitled “Redesigning classroom and experiential teaching peer evaluation tools to strengthen the peer review process”. They described the Peer assessment system as a major component in the growth and development of board members. They described that periodic peer evaluation is essential to ensure the quality of students’ education and teachers’ progress; and pointed out that considering the ceaseless change in educational methods and curricula over time, the process of peer evaluation also needs to be revised accordingly [19].

Barbara Brown and her colleague published a review study, in 1994, in Canada, entitled “The use of Peer Assessment in promoting nursing faculty teaching effectiveness: a review of the literature”. They demonstrated that the success of peer assessment depends on the educational progress of faculty members. It requires faculty participation, trained observers, short but objective methods, and constructive feedback, as well as open communication and trust between faculty members [12]. In our study, it was revealed that the success rate of peer evaluation for the advancement of faculty members’ training was higher among female faculty members, who were employed with permanent contracts, and had more than 5 years of experience. Accordingly, it seems that females along with the more experienced and permanently employed faculty members are more sensitive to professional improvement, as compared to the others.

However, it seems the online developmental model of peer observation reduces people’s resistance to face-to-face assessment and prepares them for peer assessment in face-to-face classes. What is not found in this study, and is suggested to be investigated in further research, is the value of peer evaluation for the evaluators themselves. Observing the teaching of others reveals the nuances of different teaching styles and represents the flaws that are difficult to understand during one’s own teaching. Also comparing the results of peer evaluation with those of the student evaluation and investigation of observee perception were suggested for further studies.

However, limitations of this study is the implementation only in medical basic sciences department so studies in larger sizes and other context are recommended.

## Conclusion

Virtual and online education can be a suitable platform for the implementation of faculty’s formative and developmental model of peer observation of teaching; and should be considered as an opportunity to enhance and improve the quality of virtual education.

## Electronic supplementary material

Below is the link to the electronic supplementary material.

Supplementary Material 1

## Data Availability

All completed checklist and descriptive feedback letters to faculties is available in Persian and kept confidential. Data are however available from the authors upon reasonable request and with permission. The datasets used and/or analysed during the current study available from the corresponding author on reasonable request.
