# Supplementary material for: The effects of developmental model of peer observation on the virtual teaching quality of basic medical sciences faculty
Source: BMC Med Educ. 2023 May 22;23:358. doi: 10.1186/s12909-023-04331-z (PMC10201744; doi:10.1186/s12909-023-04331-z)
Supplement: Supplementary file 1 — Supplementary Material 1 [file 12909_2023_4331_MOESM1_ESM.docx]

***Check list of peer observation of virtual classes in school of medicine***

**Observer:**

age: ……... gender: male🖵 female🖵

**Examinee:**

code: ……. age: ……... gender: male🖵 female🖵 experience in months: ……………

Employment status:………………..

**Class information:**

Number of students: …………... semester: ……… course: ………………. Date/time: ………….

| **Domain** | **Items** | **Excellent** | **Good** | **Average** | **Weak** | **Very weak** | **Not observed** |
| --- | --- | --- | --- | --- | --- | --- | --- |
| **Management of virtual class** | The goals and plan of the lesson were told at the beginning of the class. |  |  |  |  |  |  |
|  | The online class started on time and with the teacher's preparation. |  |  |  |  |  |  |
|  | The duration of teaching and the length of the class were observed according to the schedule. |  |  |  |  |  |  |
|  | The technical handling to manage the virtual class |  |  |  |  |  |  |
|  | The teacher's mastery and preparation of educational content |  |  |  |  |  |  |
|  | The attractiveness of the teacher’s presentation methods in the virtual environment |  |  |  |  |  |  |
|  | Management of contradictions and conflicts in virtual discussions |  |  |  |  |  |  |
| **Content quality** | Providing up-to-date scientific content |  |  |  |  |  |  |
|  | Adapting the content to the needs of the learners |  |  |  |  |  |  |
|  | Suitability of the presented content with the goals and topic |  |  |  |  |  |  |
|  | Proportion of the amount of content based on the course unit |  |  |  |  |  |  |
|  | Providing content tailored to learning styles |  |  |  |  |  |  |
|  | Designing presented content according instructional design principles and included images and highlights and attractive points. |  |  |  |  |  |  |
| **Organizing interactions** | Discussion and group work using virtual environment facilities during the course |  |  |  |  |  |  |
|  | Encouraging learners to interact with each other |  |  |  |  |  |  |
|  | Providing timely feedback and answering questions in a timely manner (Q&A) |  |  |  |  |  |  |
|  | Using learning material interactivity |  |  |  |  |  |  |
|  | Creating an intimate and interactive environment in the virtual classroom |  |  |  |  |  |  |
| **Motivation management** | Follow up on non-participation of students |  |  |  |  |  |  |
|  | Encouraging the student by the teacher to participate in learning |  |  |  |  |  |  |
|  | Feedback of homework results and presentation of the best student to motivate |  |  |  |  |  |  |
|  | Creating an environment for free expression of opinions |  |  |  |  |  |  |
|  | Guidance and encouragement to cooperation and collaborative works |  |  |  |  |  |  |

***Description of observation***

**Main points of observation:** ...…………………………………………………………………………………………………………………………………………………………………………………………………………………………………………………………………………………………………………………………………………………………………………………………….

...…………………………………………………………………………………………………………………………………………………………………………………………………………………………………………………………………………………………………………………………………………………………………………………………….

**Weakness:**

...…………………………………………………………………………………………………………………………………………………………………………………………………………………………………………………………………………………………………………………………………………………………………………………………….

...…………………………………………………………………………………………………………………………………………………………………………………………………………………………………………………………………………………………………………………………………………………………………………………………….

**Suggestions:**

...…………………………………………………………………………………………………………………………………………………………………………………………………………………………………………………………………………………………………………………………………………………………………………………………….

...…………………………………………………………………………………………………………………………………………………………………………………………………………………………………………………………………………………………………………………………………………………………………………………………….
